# Prevalence of Lower Back Pain in Portuguese Equestrian Riders

**DOI:** 10.3390/sports12080207

**Published:** 2024-07-30

**Authors:** Carlota Duarte, Rute Santos, Orlando Fernandes, Armando Raimundo

**Affiliations:** 1 Escola Superior de Biociências de Elvas, Instituto Politécnico de Portalegre, 7300-110 Portalegre, Portugal; carlotaduarte@ipportalegre.pt; 2 VALORIZA—Research Centre for Endogenous Resource Valorization, Instituto Politécnico de Portalegre, 7300-555 Portalegre, Portugal; 3 Escola de Saúde e Desenvolvimento Humano, Universidade de Évora, 7004-516 Évora, Portugal; orlandoj@uevora.pt (O.F.); ammr@uevora.pt (A.R.); 4 CHRC—Comprehensive Health Research Centre, Universidade de Évora, 7004-516 Évora, Portugal

**Keywords:** equestrian, lower back pain, 12-month prevalence, workload, Roland Morris disability score, body mass index, age

## Abstract

Lower back pain is prevalent in equestrian athletes, but its prevalence and associated factors are unknown in the Portuguese equestrian population. A questionnaire regarding lower back pain and possible associated factors was answered by 347 respondents. Of the respondents, 214 (61.7%) stated having experienced lower back pain in the past 12 months and therefore completed the Roland Morris disability questionnaire. Among the latter, 63.1% stated that lower back pain impaired their performance. The probability of suffering from lower back pain was higher in individuals with higher weekly riding workloads, who reported equestrianism as their main occupation, and who performed daily stable duties. Considering a Roland Morris disability score of 4 as the cut-off value for dysfunction, this sample had an average score of 5.39 ± 4.42. Individuals who stated equestrianism was their main occupation showed a significantly higher risk (OR = 1.759, *p* = 0.041) of exhibiting a score ≥ 4 than those who stated equestrianism as a hobby. Age (*p* = 0.029), body mass index (*p* = 0.047), and daily performance of stable duties (*p* = 0.030) were also associated with a higher Roland Morris disability score. Further research is needed to understand the full impacts of lower back pain in Portuguese equestrian athletes.

## 1. Introduction

Equestrian sports science is an emerging field [1] that is often based on experimental learning and tradition, instead of being centred on scientific knowledge [2]. Additionally, it has been predominately focused on the horse, while the analysis of the rider has expanded only in the last two decades [2]. To understand the various demands, dangers, and opportunities riders face, it is essential to use scientific, evidence-based investigation methods [3].

Compared with other sports, the career of equestrian athletes can be very long, with children starting highly competitive pony divisions at the ages of 5 and 6 [4] and riders competing at an Olympic level in their 60’s and 70’s [5]. As such, according to Long Term Athlete Development, equestrianism is categorized as an “early start-late specialization” sport [6]. Furthermore, horse riding is a hazardous activity, with one-fifth of all equestrians suffering serious injuries during their riding careers. Hence, research has mainly focused on acute riding injuries, but over-use injuries, repetitive strain, and lifestyle during long equestrian careers could cause chronic pain [7].

The appropriate position of the rider on the horse is “upright, balanced, elastic, solid and interactive”, the shoulder, hip, and heel should be in alignment [3], the pelvis in a neutral position, keeping a controlled upright trunk and adapting to the horse’s movements [6]. Riders with a correct riding position are most likely to achieve optimal performances but also reduce the risk of falling of the horse and possible injury [3]. The horse–rider relationship requires clear communication, and the rider must maintain balance and posture to be able to administer predictable cues (called aids) to communicate with the horse [6]. However, chronic pain may impair the rider’s balance and posture. 

Equestrian sports entail substantial and repetitive compressive mechanical forces primarily absorbed by the vertical axis of the rider’s body, notably, the lumbopelvic–hip complex [4,8,9]. The repetitive nature of training imposes significant demands on the musculoskeletal system, leading to muscle tightness [8,10], as the rider’s postural control relies heavily on coordination and neuromuscular awareness of the core and back musculature [7,11]. Although the primary cause of injury in equestrian athletes is falling of the horse [7,12], pain due to overuse or chronic injuries can diminish balance, physical performance, and sports participation, impacting athletes’ success [5,6,7]. Despite injuries and pain, equestrian athletes often continue to train and compete because of various factors, such as pressure from sponsors and horse owners [7], which in turn may contribute to aggravated injuries, impair competitive success, and compromise their overall wellbeing. Furthermore, horses are trained to recognize subtle cues; a rider overcompensating because of pain can cause training difficulties [4] and discomfort for their equine partner.

Lower back pain (LBP) can be defined as pain and/or discomfort localized below the costal margin and above the inferior gluteal folds [9]. Physical activity is both a preventive and a possible risk factor for LBP [9]. In 2020, LBP afflicted 619 million individuals worldwide, with projections suggesting an increase of up to 843 million cases by 2050, mainly due to population growth and ageing [13]. LBP is the most common chronic injury in equestrian athletes [7,8], and its incidence is higher in equestrian athletes than in other athletes and the general population [9,10]. The main risk factors that have been reported for LBP in equestrian athletes are the practice of the sport itself, because of its specific features, as described above [8], the level of expertise in the sport, consequences of acute trauma and its poor recovery [1], asymmetric posture [8,10], poor postural control [11], the cushioning and depth of the saddle seat [9,14], lack of balance, stability, and alignment at the pelvic level [10,11]. Equestrian athletes with LBP tend to have affected performance (due to distraction caused by the pain), a higher risk of falling due to earlier onset of fatigue [7,8], and a reduced ability to maintain the correct riding position and synchronize with the horse’s movements [7].

Measuring the functional outcomes in individuals with LBP has been performed using a variety of validated questionnaires, of which the most widely accepted are the Roland Morris and Oswestry questionnaires [15]. The Roland Morris disability questionnaire is a tool that enables a discriminating outcome measure in LBP [16]. It has previously been used to measure the impact of LBP on everyday functioning [17] and has been translated into several languages [15] and validated for the Portuguese population [18]. Stratford and Riddle [19] defined a threshold score of 4 in the Roland Morris disability questionnaire as a reasonably accurate value to discriminate patients according to their functionality in everyday living.

The purpose of this observational cross-sectional study is to investigate the prevalence of LBP in Portuguese equestrian athletes and to gain insight into the primary factors or possible causes leading to LBP in this population.

## 2. Materials and Methods

### 2.1. Participants

An online questionnaire was designed using LimeSurvey (https://www.limesurvey.org/pt, accessed on 10 March 2023). Participants were equestrian athletes over 18 years old, federated in the Portuguese Equestrian Federation in 2022 and/or 2023. Participation was anonymous and voluntary, and consent was given prior to opening the questionnaire. The questionnaire was disseminated using social media (Facebook, Instagram, and WhatsApp) and by asking those answering the questionnaire to share the link with other horse riders and on their social media pages (a so-called snowball sampling technique). Prior to publication, the questionnaire was submitted to a small sample of subjects and evaluated by an expert panel for validation. According to data reported by the Portuguese Equestrian Federation, in 2021, there were 8076 registered practitioners, of which about 3500 were senior-level athletes (over 18 years of age). The questionnaire was available online for two and a half months (from 10 April to 29 June 2023) when the minimum number of valid responses was obtained (347), reaching approximately 10% of the senior-level equestrian athletes enrolled in the Portuguese Equestrian Federation. The sample size was calculated with a confidence level of 95%, a confidence interval (margin of error) of 5%, and assuming a 50% response distribution using Raosoft ^®^ sample size calculator [20].

### 2.2. Questionnaire 

The questionnaire comprised 50 questions divided into 7 sections, taking approximately 10 min to complete. The first section covered demographic data (age, sex, height, and weight), while the second delved into equestrian sports practices (years of practice, weekly practice hours, and federated discipline). The third section addressed other sporting activities and routines, while the fourth focused on injuries and lower back pain, based on the questionnaire priorly used on Italian equestrians [19]. Lower back pain was defined as pain, discomfort, or numbness in the lower back area, and an accompanying illustration was provided to aid participants. The fifth section queried pain experienced during daily equestrian practices, while the sixth explored related routines and characteristics. The seventh and final section contained the Roland Morris disability questionnaire [16,18]. This tool comprises a 24-item set that patients are asked to endorse (score 1) or leave blank (score 0), and results in a total score (Roland Morris disability score, RMDS) between 0 and 24, where higher values correspond to higher levels of pain-related disability [19]. A translation of the questionnaire is presented as Supplementary Material 1.

A threshold value of 4 was considered to classify patients with LBP as functional or dysfunctional, according to a previous study that considered this threshold to provide reasonable accuracy in distinguishing between lower back pain adult patients who met their functionality goals and those who did not [19]. Sections five and seven were exclusively presented to respondents who reported experiencing LBP in the past 12 months. For further analysis, the Body Mass Index (BMI) was calculated by dividing the person’s weight, in kilograms, by their height, in meters squared; individual BMI was classified into the following categories: underweight, normal weight, overweight, and obese [21].

### 2.3. Statistical Analysis 

Statistical treatment of data was performed using SPSS version 27.0 (IBM SPSS Statistics, Armonk, NY, USA) [22]. The normality of the distribution for each continuous variable (age, height, body mass, body mass index, RMDS) was examined using the Kolmogorov–Smirnov and Shapiro–Wilk normality tests. Variables revealed a non-normal distribution and hence, non-parametric tests (Mann–Whitney U, Kruskal–Wallis, Chi-square contingency coefficient) were used. 

Regression models included univariate binary logistic models (to calculate unadjusted odds ratios for categorical variables, considering the presence or absence of LBP, and a Functional/Dysfunctional RMDS, as binary categorical outcomes), multivariate binary logistic models (to calculate adjusted odds ratios for categorical variables, considering the presence or absence of LBP, and a Functional/Dysfunctional RMDS as binary categorical outcomes), and multivariable linear regression (to calculate regression coefficients for continuous variables, considering RMDS as the dependent variable). In the multivariable models, variables were screened for independence of observations (Durbin–Watson statistic), linear relationships (observation of partial regression plots), homoscedasticity, multicollinearity, and approximate normality of residual distribution, using the multiple regression procedures in SPSS.

Taking the former regression results into account, several Generalized Linear Models (GenLM) were computed, and adjustment to the data was compared using Akaike Information Criteria (AIC) values. The GenLM that best suited the data was a main effects model that included RMDS as the dependent variable, equestrianism as the main occupation (yes/no) and daily performance of stable duties (yes/no) as predictors, and age and BMI as continuous covariates, outperforming the null (intercept) model, according to an Omnibus test (*p* = 0.002). The model considered a Gamma distribution of RMDS, and the relationship between RMDS and the predictors and covariates via a Log link function. The raw values for the covariates age and BMI were computed in the model. Estimated Marginal Means (EMMs) were adjusted for the average covariate values, as means for the reduction in the standard error due to a significant association between the covariates and the continuous dependent variable (RMDS).

## 3. Results

### 3.1. Demographics and Anthropometric Data

Of the 347 respondents, 40.1% were enrolled as show jumping riders, 22.8% as dressage riders, and 21.9% as general riders (including equestrians not involved in national competitions, rider instructors, and other officials). The remaining disciplines had minor representation within the sample. Female and male respondents represented 58.8% and 41.2%, respectively. Male respondents were older, taller, and heavier than females, and their BMI was higher, even though the average BMI fell in the normal weight category for both sexes (see Supplementary Material S2—Table S1).

In our sample, 21.3% of respondents were 35 years old or older. This proportion was 23.1% in those who considered equestrianism their main occupation, and 19.8% in those who did not, although the difference was non-significant (*p* = 0.455).

Overall anthropometric data, years of practice (means ± standard deviations) and sex (female/male) are presented in Table 1, as well as the results of a Mann–Whitney U test and a Chi-square test (for sex) that compared the group that stated that equestrianism was their main occupation with the group that stated it was not. No significant differences were found for anthropometric data, but years of equestrian practice were significantly higher (*p* = 0.004) in the group that stated their main occupation was equestrianism. Of the female respondents, 41.2% stated equestrianism was their main occupation, compared with 53.1% of the male respondents (*p* = 0.028).

### 3.2. Prevalence of Lower Back Pain in the Past 12 Months

The overall prevalence of lower back pain was 61.7% (95% confidence interval: 56.5–66.6%), with no significant differences between women and men (64.2% vs. 58.0%, *p* = 0.243). Prevalence in the group that stated equestrianism was their main occupation was not statistically different from the one presented in the group that did not (67.5% vs. 56.7%, *p* = 0.087). Among the main occupation equestrians, the prevalence of LBP showed no significant differences between women and men (67.9% vs. 67.1%, *p* = 0.914). The same was observed in the group of hobby equestrians (female prevalence: 61.7%, male prevalence: 47.8%, *p* = 0.066). A significant association between lower back pain and riding discipline could not be found (*p* = 0.590) (see Supplementary Material S2—Table S2).

### 3.3. Factors Associated with the Presence of Lower Back Pain in the Past 12 Months

The unadjusted odds ratio (OR) and 95% confidence intervals were calculated for each variable (Table 2). The occurrence of LBP in the past 12 months was associated with a higher weekly workload (*p* = 0.045), equestrianism as a primary occupation (*p* = 0.039), and daily involvement in stable duties (*p* = 0.029). There was no significant association between LBP in the past 12 months and the practice of other sports or performing warm-up exercises before riding. 

A binary logistic regression model correctly predicted 63.1% of the LBP outcomes and presented a significant Omnibus test result (*p* = 0.024). Adjusted ORs were non-significant for all variables (see Supplementary Material S2—Table S3).

As expected, the rate of individuals who rode 7 or more hours per week and who performed daily stable duties was higher in the group that considered equestrianism as the main occupation compared with the group that considered it a hobby (see Supplementary Material S2—Table S4). The results of the Mann–Whitney U tests showed no significant differences between groups with and without LBP in the past 12 months regarding age, BMI, and years of equestrian practice (see Supplementary Material—Table S5).

### 3.4. Factors Associated with the Roland Morris Disability Score (RMDS) in Individuals Who Experienced LBP in the Past 12 Months

From the 214 respondents (61.7%) who stated having felt LBP in the past 12 months and responded to the Roland Morris disability questionnaire, an RMDS of 5.39 ± 4.42 was calculated (mean ± standard deviation).

Mann–Whitney U tests and Kruskal–Wallis tests found no significant differences in RMDS due to sex (*p* = 0.304), age class (*p* = 0.309), or BMI category (*p* = 0.065) but found a significant difference in RMDS between riders that stated equestrianism was their main occupation and those that did not (means ± standard deviations: 6.10 ± 4.74 vs. 4.67 ± 3.97, *p* = 0.017). Within these respondents, 135 (63.1%) considered that LBP impaired their performance, 91 (42.5%) felt that LBP was aggravated while riding, and 58 (27.1%) and 56 (26.2%) felt that LBP was aggravated while cleaning/grooming and lunging horses, respectively, and 118 (55.1%) felt that LBP as aggravated while “mucking out” (removing manure and dirty bedding from horse stalls).

The multiple linear regression model for RMDS was statistically significant (*p* = 0.016) (Table 3), with higher BMI being associated with higher RMDS. The association among RMDS, age, and years of practice was non-significant.

The unadjusted odds ratio and 95% confidence intervals were calculated for each variable (Table 4). There was no association between a Dysfunctional score and sex, weekly riding load, daily stable duties, the practice of other sports, and warm-up exercise before riding. Nonetheless, individuals who stated that equestrianism was their main occupation showed a significantly higher risk (OR = 1.759) of an RMDS ≥ 4 (Dysfunctional) than those who did not.

A binary logistic regression model correctly predicted only 58.4% of the Dysfunctional/Functional RMDS outcomes and presented a non-significant Omnibus test result (*p* = 0.297). However, a similar model applied separately to equestrians in the main occupation and the hobby groups revealed different results in both groups. In the main occupation group, the model presented a non-significant Omnibus test result (*p* = 0.296), while in the hobby group, the same test presented a significant result (*p* = 0.018), and the model correctly predicted 65.1% of the outcomes. The adjusted OR for daily grooming duties was 4.335 (*p* = 0.001), while *p*-values for the remaining variables were non-significant (see Supplementary Material S2—Table S6).

The results of Mann–Whitney U tests showed no significant differences between Dysfunctional and Functional score groups for BMI (*p* = 0.075) and years of equestrian practice (*p* = 0.241). However, it showed significant differences in the age of both groups (average age of 28.88 years in the Dysfunctional score group vs. 25.54 years in the Functional score group, *p* = 0.022).

The effect tests of a Generalized Linear Model are summarized in Table 5, showing significant effects for performing daily stable duties (*p* = 0.030) and for the age (*p* = 0.029) and BMI (*p* = 0.047) covariates. The estimated marginal means (EMMs) for RMDS, with age and BMI fixed at mean values (27.19 years and 23.00, respectively) are shown in Table 6, revealing higher RMDS values for equestrianism as the main occupation and for daily performing of stable duties.

## 4. Discussion

### 4.1. Demographics and Anthropometrical Data

In this study, the distribution of female and male equestrian athletes (58.8% and 41.2%, respectively) was apparently more balanced than in similar studies in other countries [5,9,23]. Based on the last available report of the Portuguese Equestrian Federation [24], even though there is an overall predominance of female athletes (67.3%), there is a tendency for a reduced difference between sexes in the senior categories (riders over 20 years of age, with female athletes representing 53.7%). Our sample only included equestrians who were 18 years old or older, and this probably accounts for the balanced distribution between male and female respondents. Nevertheless, a significantly higher proportion of men considered equestrianism as their main activity.

Accordingly, reported data [24] show that 17.2% of Portuguese equestrian athletes are 36 years old or older, while 21.3% of respondents were 35 or older in our sample. This percentage rose to 23.1% in riders who considered equestrianism their main occupation. The average age of respondents was 28.20 ± 11.13 years, but the average number of years of equestrian practice was 16.92 ± 10.55, thus pointing to an early start in equestrian practice. Athletes in disciplines that engage skill (such as equestrian sports, sailing, and shooting) have longer lifespans compared with other athletes, particularly those involved in power disciplines (like boxing, weightlifting, and wrestling) [25]. Previous studies have shown that the equestrian sport does not fit a traditional Long Term Athlete Development (LTAD) model, best adapting to an “early start–late specialization” paradigm and characteristic longevity of the competitive career, even at the elite level competitions [26]. Competitive and general longevity of equestrian careers simultaneously potentiate the development of skills and expertise, but they represent an additional risk for progressive spine degeneration, resulting from repetitive trauma and physical stress on the spine [27].

Apart from the general need to avoid excessive weight because of health concerns and physical performance, equestrian athletes feel additional pressure to maintain a controlled weight because of the following two different factors: the impact of a larger body frame on equestrian performance, namely, in disciplines that convey an aesthetical, subjective judgment, such as Dressage [28], and issues associated with their equine counterpart’s welfare and performance [29]. These issues can probably contribute to justifying that, despite their expanded age span (18 to 72 years old) and the fact that to nearly half of the respondents, equestrianism was not their primary occupation, most respondents (74.1%) corresponded to normal weight or underweight categories.

### 4.2. Prevalence of Lower Back Pain in the Past 12 Months and Associated Factors

Lower back pain is highly prevalent and the leading cause of life-long disability in the adult population [13]. The previously reported prevalence of LBP in the global adult population ranged from 1.4 to 20.0% [30] with a 12-month prevalence of 38% [31]. However, the reported prevalence of LBP in athletes is higher, with a mean point prevalence of 42%, ranging from 18% to 80%, and rising to 51% when a 12-month prevalence is considered [32]. The reported values of the 12-month prevalence of LBP in equestrian athletes range from 88% to 100% [32], indicating a strong association between LBP and this sport. In this study, a 12-month prevalence of 61.7% was estimated. This value was considerably lower than those reported in previous studies [9,32], which may partially reflect the fact that half of the respondents stated that equestrianism was not their main occupation (non-professionals). In professional respondents, LBP prevalence rose to 67.5%. In this study, a significant association between LBP and riding discipline could not be found, a result consistent with some previous studies [33,34], even though a cross-sectional study in the Italian equestrian athlete population reported a higher prevalence in show jumping athletes when compared with athletes in other equestrian disciplines [9].

In the global population, risk factors for lower back pain include comorbid health conditions, increasing age, as well as obesity, smoking, lack of exercise, and other lifestyle factors [13]. In athletes, strong evidence for higher body weight and moderate evidence for high BMI as risk factors of LBP have been reported, as well as insufficient evidence to indicate age and sex as risk factors [35]. Other authors refer to a history of a previous episode of LBP, high training volumes, periods of load increase, and years of exposure to the sport as risk factors [32]. In this study, we found no significant association between sex, practising other sports, or warming up before riding and the occurrence of LBP. Previous studies have also found no connection among sex [1,9,33], practising other physical activity [9], and LBP in equestrians, even though other authors [5,11] have established that participating in other sporting activities and physical fitness can help equestrian athletes to prevent spinal injury. Age, BMI, and years of equestrian practice were not significantly different between groups with and without LBP in the past 12 months. Regarding the age of equestrians, previous studies report contradictory results, from a higher risk in younger ages [9] to a higher frequency of LBP in older riders [5]. Other authors also failed to find a connection between BMI [8,9,34], length of previous riding experience [8,9], and occurrence of LBP in equestrians.

On the other hand, the probability of suffering from LBP was higher when the weekly riding workload was 7 h or more, when equestrianism was the respondents’ main occupation, and when they performed daily stable duties. These results seem to point to an association between the workload and parallel chores involved in professional equestrian life and 12-month LBP prevalence, in line with the results found in previous studies that reported a weekly riding period greater than 5–6 h [9] and riding professionally [1] as risk factors for LBP in equestrian athletes. Stable duties like “mucking out”, “preparing the bedding”, and sweeping involve a bent and twisted back position most of the time, creating harmful stress loads of the posture [36] and contributing to an increased risk of musculoskeletal problems [37]. In previous studies, riders stated that stable duties were responsible for their pain [5]. To the authors’ knowledge, there is only one study regarding the injury of equestrians in Portugal. Although that study comprised all injuries, and not exclusively LBP, it reported a significant association between the occurrence of injury and the number of days of training per week, years of experience, height and weight of the rider, and practice of another sport [38].

### 4.3. Roland Morris Disability Score and Associated Factors

To our knowledge, no previous studies have specifically investigated RMDS in equestrian athletes, thus limiting the comparability of the results in this study and suggesting caution in their interpretation. Nonetheless, lower average RMDS have been reported in elite athletes from different sports, in individuals with apparently similar characteristics [39]. The RMDS was previously used to evaluate long-term functional results of equestrians who suffered spinal fractures [40], which reported an average score of 5.5 and a significant correlation between occupational disability and RMDS. In this study, the RMDS of respondents who stated equestrianism as their main occupation was significantly higher than that of those who considered it a hobby.

The results of the GenLM point to a significant effect of age (*p* = 0.029), BMI (*p* = 0.047), and daily performance of stable duties (*p* = 0.030) on the RMDS. As previously discussed, in athletes in general, there is insufficient evidence indicating that age is a risk factor for LBP [35], and some authors reported high training volumes and years of exposure to the sport as risk factors [32]. Our results, on the other hand, point to a significant effect of age but found no significant effects of the latter variables. We suggest that the longer lifespan of equestrians, when compared with other athletes, may have played a part in these results. The importance of BMI as a predictor for LBP has been previously reported in the general population [41,42], and our results suggest that appropriate BMI management is probably important in reducing LBP prevalence in equestrian athletes. As for the effect of performing daily stable duties, our results point to this being a frequent activity for Portuguese equestrians (61.4% of respondents), and even more so for those who considered equestrianism as their main occupation (73.1%). We also reported that within the group that considered equestrianism a hobby, the risk of an RMDS > 4 (dysfunctional) was more than 4-fold greater when daily stable duties were performed. In future works, it would be useful to introduce a measure of the physical fitness of respondents to further investigate risk factors for reduced functionality in equestrians.

Validation of an RMDS of 4 as a threshold in discriminating functionality in athletes and equestrians is advisable, as functionality goals in these populations are probably very different from those regarding LBP patients in the general population. It should also be noted that the average RMDS in the respondents experiencing LBP in the past 12 months was higher than this threshold, pointing to a relevant impact of LBP on the respondents’ quality of life, ability to perform their occupational duties, and competitive performance. Considering the sportive longevity of equestrian athletes [24], it would be beneficial to conduct further research regarding the causes of LBP, as well as strategies that could help its prevention or mitigation, such as specially designed training programs, as the benefits of specific exercise in preventing and improving LBP in athletes have been established in other sports [43].

### 4.4. Limitations of This Study

Although the questionnaire in this study was validated prior to its publication, it was not properly validated for the population in question. The fact that it was conducted online, with no additional contact with respondents, and no measure of motivations for response, raises the possibility of some bias in the responses. Regarding the Roland Morris disability questionnaire, previous work suggests there is no difference in scores retrieved in online and paper-based responses [44]. However, the only published results found by the authors that used RMDS as a measure of the functional outcome in equestrians [40] concerned patients who suffered spinal fractures, and hence were otherwise clinically followed up, allowing for additional validation. Another limitation is the use of the cut-off value of 4 as a measure of the functional impairment of the respondents since this threshold was established in a population of adult LBP patients undergoing physiotherapy. The authors acknowledged the need for cross-validation of this estimate and evaluation of the stability in the estimated value in people with diverse functional demands [19]. These limitations suggest that the obtained results should be interpreted with caution. In addition, the reported associations cannot be considered causations.

## 5. Conclusions

For the first time, this study presents the prevalence of lower back pain (LBP) in an apparently representative sample of Portuguese equestrian athletes. A 12-month prevalence of 61.7% was estimated. The probability of suffering from LBP was higher in individuals with higher weekly riding workloads, who reported equestrianism as their main occupation, and who performed daily stable duties. To measure the impact of LBP on the daily functioning of equestrian athletes, we used the Roland Morris disability questionnaire and calculated an average score of 5.39 ± 4.42 (mean ± standard deviation). Among the riders who experienced LBP in the past 12 months, 63.1% considered that it impaired their performance, 42.5% felt that LBP was aggravated while riding, and 55.1% felt that LBP was aggravated while removing manure and bedding from horse stalls. Significant associations among RMDS, age, BMI and the daily performance of stable duties were found. Estimated marginal means, controlling for age and BMI, showed higher scores in equestrians who were involved in daily stable duties. Further investigation should be conducted on the pain assessment of LBP in equestrian athletes and its effects on functionality.

## Figures and Tables

**Table 1:** Anthropometric data and years of practice (mean ± S.D.) and comparison of respondent status (main occupation: yes/no) (*p*-value for Mann–Whitney U test, and for Chi-square test for sex).

	Total (*n* = 347)	Equestrianism as Main Occupation		*p*-Value
		Yes (*n* = 160)	No (*n* = 187)	
Age (years)	28.20 ± 11.13	28.36 ± 10.29	28.07 ± 11.83	0.498
Height (cm)	169.71 ± 8.93	170.46 ± 8.91	169.07 ± 8.91	0.052
Weight (kg)	66.94 ± 12.74	67.79 ± 12.15	66.20 ± 13.21	0.166
BMI (kg/m^2^)	23.12 ± 3.28	23.23 ± 3.10	23.03 ± 3.44	0.461
Years of practice	16.92 ± 10.55	18.09 ± 9.68	15.91 ± 11.16	0.004
Sex (female/male)	204/143	84/76	120/67	0.028

**Table 2:** Odds ratios for factors associated with LBP in the past 12 months.

*n* = 347	Lower Back Pain in the Past 12 Months		Odds Ratio	95% Confidence Interval	*p*-Value
	Yes	No			
Female/male	131/83	73/60	1.297	0.837–2.011	0.245
Rides 7 h or more per week/up to 6 h per week	105/109	50/83	1.599	1.028–2.487	0.045
Main occupation/hobby	108/106	52/81	1.587	1.023–2.463	0.039
Daily stable duties (yes/no)	141/73	72/61	1.636	1.051–2.548	0.029
Other sports (yes/no)	114/100	80/53	0.755	0.487–1.171	0.210
Warm-up before riding (yes/no)	40/174	17/116	1.569	0.849–2.899	0.151

The reference category for each variable is underlined.

**Table 3:** Multiple linear regression analysis for RMDS.

	B (SE)	Beta	t	*p*-Value
Age	0.058 (0.047)	0.134	1.235	0.218
BMI	0.204 (0.097)	0.149	2.112	0.036
Years of practice	−0.003 (0.049)	−0.007	−0.066	0.948

B: unstandardized regression coefficient. SE: standard error.

**Table 4:** Odds ratio for factors associated with Dysfunctional/Functional RMDS.

*n* = 214	Roland Morris Disability Score (RMDS)		Odds Ratio	95% Confidence Interval	*p*-Value
	Dysfunctional (RMDS > 4)	Functional (RMDS ≤ 4)			
Female/Male	60/46	71/37	0.6797	0.3911–1.1.181	0.171
Rides 7 h or more per week/up to 6 h per week	56/50	49/59	1.349	0.788–2.309	0.276
Main occupation/hobby	61/45	47/61	1.759	1.024–3.023	0.041
Daily stable duties (yes/no)	76/30	65/43	1.676	0.946–2.969	0.077
Other sports (yes/no)	54/52	60/48	0.831	0.485–1.422	0.499
Warm-up before riding (yes/no)	20/86	20/88	1.023	0.515–2.035	0.948

Reference category for each variable is underlined.

**Table 5:** Parameter estimates for the GenLM main effects model.

Parameter	B (SE)	95% Wald Confidence Interval	Wald Chi-Square	df	*p*-Value
Intercept	0.583 (0.401)	−0.203–1.369	2.116	1	0.146
Equestrinism as main occupation (yes/no)	−0.146 (0.102)	−0.347–0.054	2.044	1	0.153
Daily stable duties (yes/no)	0.249 (0.115)	0.025–0.474	4.728	1	0.030
Age	0.011 (0.005)	0.001–0.021	4.761	1	0.029
BMI	0.032 (0.016)	<0.001–0.063	3.930	1	0.047

B: unstandardized regression coefficient. SE: standard error. df: degrees of freedom.

**Table 6:** Estimated marginal means (EMMs) for the Roland Morris disability score (RMDS), with age fixed at 27.19 years and BMI fixed at 23.00.

Predictor	Groups	EMM for RMDS	95% Confidence Interval
Overall estimated mean (*n* = 214)		5.31	4.78–5.90
Equestrianism as main occupation	Yes (*n* = 108)	5.71	4.93–6.63
	No (*n* = 106)	4.93	4.28–5.69
Daily stable duties	Yes (*n* = 141)	6.01	5.32–6.80
	No (*n* = 73)	4.69	3.92–5.61

## Data Availability

The raw data supporting the conclusions of this article will be made available by the authors upon request.

## Supplementary Materials

The following supporting information can be downloaded at: https://www.mdpi.com/article/10.3390/sports12080207/s1, Supplementary Material 1—Questionnaire; Supplementary Material 2: Table S1: Demographic and anthropometric data of respondents, according to sex; Table S2: Distribution of respondents that reported feeling Lower Back Pain in the last 12 months according to the equestrian discipline Pearson’s Chi-square and *p*-value; Table S3: Binary logistic regression model for 12-month Lower Back Pain (variables in the equation); Table S4: Odds ratio for rider status (main occupation vs. hobby), weekly riding workload and daily stable duties; Table S5: Age, BMI and years of equestrian practice—comparison of groups with, and without Lower Back Pain in the last 12 months (*p*-value for Mann-Whitney U test); Table S6: Binary logistic regression model for Dysfunctional RMDS according to rider status (main occupation vs. hobby) (variables in the equation).
