# Supplementary material for: Prevalence of Lower Back Pain in Portuguese Equestrian Riders
Source: Sports (Basel). 2024 Jul 30;12(8):207. doi: 10.3390/sports12080207 (PMC11359416; doi:10.3390/sports12080207)
Supplement: Supplementary file 1 [file sports-12-00207-s001.zip › Supplementary Material 1.pdf]

Supplementary Material 1 – Questionnaire

Objectives

To characterize equestrian athletes in Portugal, and understand the incidence of low back pain, and associated injuries, in this population.

Inclusion criteria:

- Portuguese nationality
- Age equal or over 18 years old
- Having federated license in the Portuguese equestrian federation in the years of 2023 or 2022.

Informed consent

In the scope of the PhD in Human Motricity, a study is under development, to characterize Portuguese equestrian athletes, and the rates of low back pain and injuries in this population.

Your participation is requested, by filling out a brief form, with a total duration of 10 minutes. There are no right or wrong answers, but it is important for you to read carefully, and answer honestly to every question. If you make a mistake filling out the form, it is possible for you to go back and correct your answer, but once submitted it will not be possible for you to reopen or fill out a new form.

There are 51 questions in this form.

1 – Athlete characterization

(in this section there will be questions regarding anthropometric and demographic details about the athlete)

1.1 – Age (years)*

(Please write down your answer)

1.2 – Sex: *

(Please select the one that applies)

	Feminine
	Masculine

1.3 – Height (cm)*

(Please write down your answer)

1.4 – Weight (kg)*

(Please write down your answer)

1.5 – Nationality* (Please select one answer)

	Portuguese
	Other

1.6 – Country of residence and equestrian practice *

(Please select all that apply)

	Portugal
	Spain
	Netherlands
	Germany
	United Kingdom
	United States of America
	Other

1.7 – If the country of residence and equestrian practice selected was Portugal, city of location of the equestrian center where you practice your equestrian activities. *

(Please write down your answer)

2 - Equestrian practices

(in this section there will be questions related only to your equestrian practices/ activities)

2.1 – For you equestrianism is: *

(Please select the one that applies)

	Hobby
	Profession

(in case you are in an equestrian related study program select the option “profession”)

2.2 – Years of equestrian practice*

(Please write down your answer)

2.3 – Hours of equestrian practice per week (hours/week)*

(Please select the one that applies)

	1 to 2
	3 to 4
	5 to 6
	7 to 12
	13 to 18
	More than 19

(How many hours per week you ride **horses** - in case of doubt, think about the last year 2022-2023 to answer this question)

2.4 – Equestrian discipline:*

(Please select all that apply)

	General equitation
	Dressage
	Show Jumping
	Eventing

	Endurance
	Other

You indicated that you practiced “other” equestrian discipline, which one(s)?

(Please write down your answer)

2.4.1 – Level of competition in Dressage *

(Please select the appropriate position for each element)

	Throughout life	In the last 12 months	Never
Festival			
National			
International			
Olympic			

2.4.2 – Level of competition in Show Jumping *

(Please select the appropriate position for each element)

	Throughout life	In the last 12 months	Never
Festival			
National			
International			
Olympic			

2.4.3 – Level of competition in Eventing *

(Please select the appropriate position for each element)

	Throughout life	In the last 12 months	Never
National			
International			
Olympic			

2.4.4 – Level of competition in Endurance *

(Please select the appropriate position for each element)

	Throughout life	In the last 12 months	Never
Festival			
National			
International			
Olympic			

2.4.4 – Level of competition in “Other” *

(Please select the appropriate position for each element)

	Throughout life	In the last 12 months	Never
Festival			
National			
International			

2.5 – Discipline in which you were federated in the sportive year of 2022 or 2023.

(In case you are already federated in the present year, 2023, answer regarding this year) *

(Please select all that apply)

	General equitation
	Dressage
	Show Jumping
	Eventing
	Endurance
	Other

2.6 – Sporting license in the year 2022 or 2023:*

(Please select all that apply)

	Practitioner license
	National competition license
	International competition license

2.7 – Beyond equestrian practice, do you also participate in activities inherent to treatment and management of horses?*

(Please select the one that applies)

	Yes, daily
	Yes, sometimes
	No

(in case of doubt, think about the last year 2022-2023 to answer this question)

With treatment and management of horses we mean:

- Cleaning and mucking out stalls
- Grooming, tacking and untacking horses
- Feeding of horses
- Management and maintenance of equestrian spaces
- Others

3 – Sporting practices

(in this section you should answer about your practice of other sports not related with equestrian sports)

3.1 – Other than equitation, do you practice other sports or physical activities? *

(Please select the one that applies)

	Yes
	No

3.1.1 – Do you practice other sports or physical activities to complement equitation?

(Please select the one that applies)

	Yes
	No

3.1.1.1 – If so, was it prescribed by a specialist?

(Please select the one that applies)

	Yes
	No

3.1.2 – Which sports do you practice?

(Please select all that apply)

	Gym (free training)
	Gym (personalized training)
	Walking
	Running
	Cycling
	Swimming
	Yoga
	Pilates
	Other

3.1.3 – Do you consider equitation to be your main sport?

(Please select the one that applies)

	Yes
	No

Which is your main sport?

(Please write down your answer)

3.1.4 – The other sports your practice are federated?

(Please select the one that applies)

	Yes
	No

3.1.5 – How many hours per week do you practice other sports?

(Please select the one that applies)

	1 to 2
	3 to 4
	5 to 6
	7 to 12
	13 to 18
	More than 19

4 – Musculoskeletal injuries

(In this section you will answer to questions regarding musculoskeletal injuries)

4.1 – Have you ever sustained a bone fracture?*

(Please select the one that applies)

	Yes
	No

(if you ever sustained a bone fracture diagnosed by a specialist physician)

4.1.1 – In which body region?

(Please select all that apply)

	Head
	Upper limbs and scapular waist
	Lower limbs and pelvic waist
	Spine
	Sacrum
	Coccyx
	Other

4.1.2 – Have you sustained any fractures due to the practice of equestrian sports?

(Please select the one that applies)

	Yes
	No

(Consider fractures sustained as a direct consequence of falling of a horse, or being on a horse)

4.1.3 – Have you sustained any fractures due to the direct interaction with horses?

(Please select the one that applies)

	Yes
	No

(Consider fractures sustained as a direct consequence of any activity or interaction with horses. This question does not apply to falling of a horse)

4.2 – Considering the last 12 months, have you had any problem (such as pain, discomfort or numbness) in the lower back?*

(Please select the one that applies)

	Yes
	No

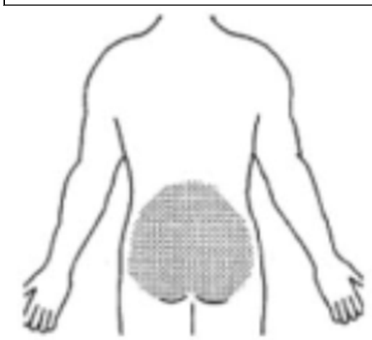

Figure 1 - The image represents the approximate region of the body mentioned in this question.

5 – Low Back Pain in equestrian practices

(Answer this category thinking about what you feel when you are developing equestrian activities)

(Answer the following questions regarding what you feel/ do when the **pain in the lower back in present)**

5.1 – Do you feel the pain is worst/ feel more pain when you ride a horse?

(Please select the one that applies)

	Yes
	No
	Never thought about that

(with "ride a horse" we mean the direct practice of equitation, of being on a horse in the three gaits)

5.2 – Do you feel that pain harms your performance while riding?

(Please select the one that applies)

	Yes
	No

5.3 – When you are on a horse when is the pain you feel stronger?

(Please select all that apply)

	Walk
	Rising trot/ posting trot
	Sitting trot
	Canter
	Canter in jumping position
	During jumps
	Does not apply

Aswer thinking about the circumstance when you feel more pain, or the pain gets worst, it is possible to choose more than one option, think about your answer.

Explanation:

- "Canter" – sitting on the saddle
- "Canter in jumping position" – cantering with short stirrups in a jumping position, with seat off the saddle.
- "During jumps" – in show jumping lessons, when you have to use the jumping position a lot over jumps.

5.4 – During horse grooming, in which situation do you feel more pain?

(Please select the one that applies)

	Cleaning the horse's body
	Cleaning the horse's hoofs
	During both
	Pain does not enhance

5.5 – When you are lunging a horse, do you feel discomfort or pain?

(Please select the one that applies)

	Yes
	No
	Does not apply to me

5.6 – Do you feel pain cleaning or mucking out stalls?

(Please select the one that applies)

	Yes
	No
	Does not apply to me

5.7 – When you ride a horse, you feel pain:

(Please select the one that applies)

	Gets worst
	Is constant
	Gets worst in the beginning, but with the continuation of exercise it returns back to normal levels
	Decreases

5.8 – When you feel pain do you do any exercise of movement that helps reduce it? If you do, what do you do?

(Please write down your answer)

5.9 – The pain you feel is constant, or accentuates in a specific season?

(Please select all that apply)

	Is constant
	Accentuates with cold
	Accentuates with humidity
	Accentuates with heat
	Other

With cold, humidity or heat we are referring to climatic conditions.

6 – Day to day in equestrianism, practices and pain

(to answer the following questions, think about what you felt/ did during the last year)

6.1 – In your daily activities do you warm up before riding on a horse?

(Please select the one that applies)

	Yes
	No

(warm up of the horse rider before riding on the horse)

6.2 – During the management of equestrian spaces/ infrastructures can you lift weights?

	Yes, without a problem
	No, some are hard to lift

6.2.1 – Which one’s can´t you lift? (answer this question only if the following are true)

(Please select all that apply)

	Wheelbarrow (with content inside)
	Poles and jumps
	Shaving bales

	Hard feed bags
	Other (things I consider heavy)
	Other (things I consider light)

“Other (things I consider heavy)” – things that are possible to lift without pain, but due to pain you can’t.

6.3 – Do you tend to wear clothes that keep the lower back region warm when you are riding horses?

(Please select the one that applies)

	Yes
	No
	Never thought about that

(example: vests, jackets or long sweaters/shirts that do not expose the lower back)

6.4 – What shoes do you use more frequently in your day to day outside of equitation?

(Please select all that apply)

	High heels
	Flat heels with rigid soles
	Sports shoes
	Orthopedic shoes

6.5 – How do you mount on the horse? (Please select all that apply)

	With a step
	With the help of someone “leg up”
	Without any help and directly from the floor
	Other

(The act of getting on the horse)

6.6 – What type of saddle do you use?

(Please select the appropriate position for each element)

	Never (0%)	Rarely (till 10%)	Frequently (more than 50%)	Always (99%)
Dressage				
Jumping/ eventing				
Mixed saddle				
Portuguese or Spanish saddle				
Endurance				

(The percentages are according to the amount of times you ride horses. Think about the last year of equestrian practice)

Example of the answer of a instructor, horse trainer and eventer below:

	Never (0%)	*Rarely (till 10%)*	*Frequently (more than 50%)*	*Always (99%)*
Dressage			*X*	
Jumping/ eventing			*X*	
Mixed saddle		*X*		
Portuguese or Spanish saddle	*X*			
Endurance	*X*			

6.7 – The saddles you use personalized and measured for you? (Please select all that apply)

	Yes (jumping saddle)
	Yes (dressage saddle)
	No

(To answer this question, think about the saddles you use more frequently. Not saddles you do not use often)

7 - The Roland-Morris Disability Questionnaire

When your back hurts, you may find it difficult to do some of the things you normally do. This list contains sentences that people have used to describe themselves when they have back pain. When you read them, you may find that some stand out because they describe you today. As you read the list, think of yourself today. When you read a sentence that describes you today, put a tick against it. If the sentence does not describe you, then leave the space blank and go on to the next one. Remember, only tick “yes” the sentence if you are sure it describes you today, if not answer “no”.

	Yes	No
I stay at home most of the time because of my back.		
I change position frequently to try and get my back comfortable.		
I walk more slowly than usual because of my back.		
Because of my back I am not doing any of the jobs that I usually do around the house.		
Because of my back, I use a handrail to get upstairs.		
Because of my back, I lie down to rest more often.		
Because of my back, I have to hold on to something to get out of an easy chair.		
Because of my back, I try to get other people to do things for me.		
I get dressed more slowly then usual because of my back.		
I only stand for short periods of time because of my back.		
Because of my back, I try not to bend or kneel down.		
I find it difficult to get out of a chair because of my back.		
My back is painful almost all the time.		
I find it difficult to turn over in bed because of my back.		
My appetite is not very good because of my back pain.		
I have trouble putting on my socks (or stockings) because of the pain in my back.		
I only walk short distances because of my back.		
I sleep less well because of my back.		
Because of my back pain, I get dressed with help from someone else.		
I sit down for most of the day because of my back.		
I avoid heavy jobs around the house because of my back.		
Because of my back pain, I am more irritable and bad tempered with people than usual.		
Because of my back, I go upstairs more slowly than usual.		
I stay in bed most of the time because of my back.		
