# Supplementary material for: Prevalence of Lower Back Pain in Portuguese Equestrian Riders
Source: Sports (Basel). 2024 Jul 30;12(8):207. doi: 10.3390/sports12080207 (PMC11359416; doi:10.3390/sports12080207)
Supplement: Supplementary file 1 [file sports-12-00207-s001.zip › Supplementary Material 2.pdf]

Supplementary Material 2

Table S1: Demographic and anthropometric data of respondents, according to sex.

**Discipline[1]**		**Dressage**	**Jumping**	**Eventing**	**General**	**Endurance**	**Horseball**	**Working Equitation**	**Total**
n (%)		79 (22.8)	139 (40.1)	30 (8.6)	76 (21.9)	14 (4.0)	5 (1.4)	4 (1.2)	347 (100)
Female	*n* (%)	44 (21.6)	86 (42.2)	7 (3.4)	51 (25.0)	9 (4.4)	5 (2.5)	2 (1.0)	204 (100)
Male	*n* (%)	35 (24.5)	53 (37.1)	23 (16.1)	25 (17.5)	5 (3.5)	0 (0)	2 (1.4)	143 (100)
Age (years)	Female	23.7	30.9	24.7	26.7	24.9	22.0	22.5	24.8
	Male	28.4	40.1	36.8	32.9	32.6	---	28.0	33.0
Height (cm)	Female	165	168	165	163	163	167	164	165
	Male	177	178	176	178	173	---	178	177
Weight (kg)	Female	59.7	59.4	61.2	60.5	59.0	60.8	57.5	60.2
	Male	74.1	79.9	77.0	78.0	72.0	---	81.0	76.5
BMI	Female	21.9	21.0	22.6	22.7	22.2	21.7	21.3	22.2
	Male	23.7	25.3	24.7	24.6	23.9	---	25.6	24.4

1 – According to the last National Federation inscription

Table S2: Distribution of respondents that reported feeling Lower Back Pain in the last 12 months according to the equestrian discipline Pearson's Chi-square and p-value.

**12 month LBP**	**Dressage**	**Endurance**	**Eventing**	**General**	**Horseball**	**Jumping**	**Working Equitation**	**Total**
Yes	53	9	18	51	3	77	3	214
No	26	5	12	25	2	62	1	133
Total	79	14	30	76	5	139	4	347

	**Value**	**Degrees of freedom**	***p*-Value**
**Pearson's Chi-square**	4.629	6	0.592

Table S3: Binary logistic regression model for 12-month Lower Back Pain (variables in the equation)

	**B (SE)**	**Adj. OR**	**95% Confidence Interval**	***p*-Value**
Sex (female/male)	0.399 (0.240)	1.490	0.931 - 2.385	0.096
Main occupation (no/yes)	-0.264 (0.258)	0.768	0.463 - 1.274	0.308
Daily stable duties (yes/no)	0.365 (0.238)	1.441	0.904 - 2.296	0.124
Other sports (no/yes)	0.219 (0.234)	1.245	0.786 - 1.971	0.350
Warm up (no/yes)	-0.556 (0.328)	0.573	0.301 - 1.090	0.090
Weekly workload (up to 6h/7h or more)	0.304 (0.263)	1.355	0.809 - 2.270	0.249
Constant	0.109 (0.583)	1.115		0.852

B: unstandardized regression coefficient. SE: standard error. Adj. OR: adjusted odds ratio. Reference category for each variable is underlined.

Table S4: Odds ratio for rider status (main occupation vs. hobby), weekly riding workload and daily stable duties.

	Main occupation	Hobby	Odds Ratio	95% Confidence Interval	*p*-Value
Weekly load: 7h or more/up to 6 h	111/49	44/143	7.3622	4.5710 - 11.8580	< 0.0001
Daily stable duties: yes/no	117/43	96/91	2.5792	1.6411 - 4.0536	< 0.0001

Reference category for each variable is underlined.

Table S5: Age, BMI and years of equestrian practice – comparison of groups with, and without Lower Back Pain in the last 12 months (p-value for Mann-Whitney U test).

	12 month LBP – Yes (*n* = 214)	12 month LBP – No (*n* = 133)	*p*-Value
Age (years)	27.16 ± 10.31	29.83 ± 12.24	0.061
BMI (kg/m^2)	22.95 ± 3.23	23.40 ± 3.37	0.178
Years of equestrian practice	16.28 ± 9.92	17.93 ± 11.45	0.245

Table S6: Binary logistic regression model for Dysfunctional RMDS according to rider status (main occupation vs. hobby) (variables in the equation)

Group	Variables	B (SE)	Adj. OR	95% Confidence Interval	*p*-Value
Hobby	Sex (female/male)	-0.821 (0.485)	0.440	0.170 - 1.140	0.091
	Daily stable duties (yes/no)	1.467 (0.457)	4.335	1.770 - 10.621	**0.001**
	Other sports (no/yes)	0.216 (0.441)	1.241	0.522 - 2.948	0.625
	Warm up (no/yes)	0.586 (0.572)	1.796	0.586 - 5.511	0.306
	Weekly workload (up to 6h/7h or more)	-0.553 (0.510)	0.575	0.212 - 1.564	0.279
	Constant	-0.394)0.874)	0.674		0.652
Main occupation	Sex (female/male)	-0.091 (0.425)	0.913	0.397 - 2.102	0.831
	Daily stable duties (yes/no)	-0.852 (0.536)	0.427	0.149 - 1.219	0.112
	Other sports (no/yes)	0.089 (0.419)	1.093	0.481 - 2.487	0.831
	Warm up (no/yes)	-0.286 (0.551)	0.751	0.255 - 2.212	0.603
	Weekly workload (up to 6h/7h or more)	0.024 (0.457)	1.024	0.418 - 2.510	0.959
	Constant	1.146 (1.036)	3.146		0.269

B: unstandardized regression coefficient. SE: standard error. Adj. OR: adjusted odds ratio. Reference category for each variable is underlined.
